# LED Lighting Strategies Affect Physiology and Resilience to Pathogens and Pests in Eggplant (*Solanum melongena* L.)

**DOI:** 10.3389/fpls.2020.610046

**Published:** 2021-01-13

**Authors:** J. Anja Dieleman, H. Marjolein Kruidhof, Kees Weerheim, Kirsten Leiss

**Affiliations:** Business Unit Greenhouse Horticulture, Wageningen University & Research, Wageningen, Netherlands

**Keywords:** Botrytis cinera, far-red light, blue light, shade avoidance, defense response, *Solanum melongena* (L.)

## Abstract

Over the last decade, LED lighting has gained considerable interest as an energy-efficient supplemental light source in greenhouse horticulture that can change rapidly in intensity and spectral composition. Spectral composition not only affects crop physiology but may also affect the biology of pathogens, pests, and their natural enemies, both directly and indirectly through an impact on induced plant resistance. In this study, we investigated the effects of light spectrum against a background of sunlight on growth and development of *Solanum melongena*. These effects were related to the spectral effects on the establishment of populations of the predatory mite *Amblyseius swirskii* and plant resilience against the biotrophic fungus powdery mildew, the necrotrophic fungus botrytis, and the herbivorous arthropod Western flower thrips. The effects of a reduced red/far-red (R:FR) ratio were studied under two ratios of red to blue light. Far-red light either was supplied additionally to the photosynthetic photon flux density (PPFD) or partially replaced PPFD, while maintaining total photon flux density (PFD). Effects of white light or additional UV-B light on plant resilience was tested, compared to the reference (5% blue, 5% green, and 90% red light). Plant biomass in the vegetative phase increased when additional far-red light was supplied. Stem length increased with far-red, irrespective of PPFD and the percentage of blue light. In the generative phase, total shoot biomass and fruit fresh weights were higher under additional far-red light, followed by the treatments where far-red partly replaced PPFD. Far-red light increased biomass partitioning into the fruits, at the expense of the leaves. There were no differences in population growth of *A. swirskii* mites between light treatments, nor did light treatment have an effect on the vertical distribution of these predatory mites in the plants. The treatments with additional far-red light reduced the infection rate of powdery mildew, but increased botrytis infection. These differences might be due to the plant defenses acting against these pathogens evolving from two different regulatory pathways. These results show that positive effects of altered spectral compositions on physiological responses were only moderately compensated by increased susceptibility to fungal pathogens, which offers perspective for a sustainable greenhouse horticulture.

## Introduction

Light is considered to be the most important environmental factor affecting plant development, growth, and production in greenhouse horticulture. In northern latitudes, solar light levels during the winter can be insufficient to maintain production levels and product quality, due to the low light intensities and short photoperiods (Davis and Burns, 2016). The potential of supplemental lighting to foster off-seasonal production in the Mediterranean region is also now under investigation (Palmitessa et al., 2020; Paucek et al., 2020). Under low-light conditions, natural light can be supplemented by artificial lighting, with high pressure sodium (HPS) fixtures currently being the predominant greenhouse lighting source. However, the introduction of LED lighting systems has received considerable attention over the last decade. Benefits of LED lighting are the high efficiency with which they convert electricity into light, low heat emission, and long lifetime. Where HPS emits a fixed spectrum of approximately 4% blue, 34% green, 50% red, and 12% far-red light, LEDs can emit narrow-bandwidth light allowing the design and optimization of a dedicated light spectrum for plant growth and development (Morrow, 2008). However, before the full potential of LEDs as light source for plant production in greenhouses can be used, plant responses to spectral composition of the light as well as the effects on the biology of pathogens, pests, and their natural enemies have to be quantified.

Red photons are well absorbed by leaves, photosynthetically highly efficient (McCree, 1972), and efficiently generated by LEDs. Therefore, red is the basis light color in most commercially used light sources in protected cultivation of plants (Kusuma et al., 2020). Some blue light (ranging from 5 to 10%) is typically added to improve growth and prevent excessive stem elongation (Hernández and Kubota, 2016). Adding far-red light to a red/blue light spectrum was recently shown to increase fruit production in fruit vegetable crops such as tomato (Ji et al., 2019; Kalaitzoglou et al., 2019). A decreased ratio of red to far-red light by adding far-red light to the spectrum initiates shade avoidance symptoms, leading to increased internode and petiole elongation (Franklin and Quail, 2010), upward leaf movement (hyponasty; Keuskamp et al., 2010), reduced branching (Finlayson et al., 2010), and accelerated flowering, as reviewed by Demotes-Mainard et al. (2016). The positive effect of a reduced red/far-red ratio on plant biomass may be explained by an increase in light interception due to the altered plant architecture (Heraut-Bron et al., 2001; Sarlikioti et al., 2011), increased rate of photosynthesis (Zhen and van Iersel, 2017; Zhen and Bugbee, 2020), and biomass partitioning in favor of the generative plant parts (Ji et al., 2020). To which extent the addition of far-red light has comparable effects in other fruit vegetable crops such as eggplant (*Solanum melongena*) still has to be determined. In most studies, positive effects of far-red light were established by giving far-red additional to the PAR light spectrum. However, this implies that the total photon flux density (PFD) is increased and thereby also the electricity consumption of the LED lighting system. From a sustainability perspective, the question would be whether comparable effects of far-red light can be achieved by replacing part of the PAR light by far-red, thereby maintaining the PFD and, under the assumption that photons with different wavelengths are produced equally efficient, the energy input in the greenhouse system. Recent results have shown that far-red photons are equally efficient at driving canopy photosynthesis when acting synergistically with traditionally defined photosynthetic photons (Zhen and Bugbee, 2020). This opens the discussion to which extent the definition of PAR should be adjusted, incorporating wavelengths up to 750 nm. In contrast to the effects of far-red, blue light reduces plant height and leaf expansion in nearly all species (Huché- Thélier et al., 2016; Dieleman et al., 2019). Runkle and Heins (2001) showed that an environment deficient in blue promoted stem extension in all long day species tested, independent of the red/far-red ratio. This offers perspectives to suppress an expected stem elongation as a consequence of a low red/far-red ratio by decreasing the red/blue ratio. Increasing the proportion of blue light increases the rate of leaf photosynthesis (Hogewoning et al., 2010), which might be related to the increased concentration of photosynthetically active pigments such as chlorophyll (Meng and Runkle, 2019). To which extent an increased proportion of blue light compensates for far-red-mediated plant responses in eggplant remains to be established.

The spectral composition of the light not only influences crop growth and development but also affects the biology of pathogens, pests, and their natural enemies. Until recently, however, these effects have rarely been investigated. Light spectrum can influence pests and pathogens directly or indirectly, via host plant resistance. UV-B, for example, is almost absent in most of the light spectra to which greenhouse crops are exposed, as this part of the natural sunlight spectrum is largely filtered out by most cladding materials. The response of plants to UV-B is regulated by a UV-B-specific photoreceptor called UV RESISTANT LOCUS (UVR8). Apart from steering the expression of genes that code for inhibition of hypocotyl elongation and DNA repair, UVR8 also codes for genes involved in antioxidative defenses and the production of phenols that can play a role in plant defense against pests and diseases (Rizzini et al., 2011; Escobar-Bravo et al., 2017). Indeed, several studies indicate that deficiency in UV-B in greenhouse environments can lead to increased susceptibility of plants for pests and diseases (Caldwell et al., 2003; Ballaré et al., 2011; Kuhlmann and Muller, 2011; Demkura and Ballaré, 2012; Ballaré, 2014; Huché- Thélier et al., 2016). In addition to short-wavelength UV-B light, light with longer wavelengths can also influence plant defenses against pathogens and pests. Young cucumber plants grown under red monochromatic LEDs, for example, had lower incidence of powdery mildew than plants grown under white and other monochromatic lights (Wang et al., 2013). Moreover, monochromatic red and blue LEDs inhibited *B. cinerea* infection in grapevine through an increase of plant defense-related stilbenes (Ahn et al., 2015).

These effects of light on plant resistance show that defense pathways interact with and are influenced by light-dependent processes (Kangasjärvi et al., 2012), whereby light has emerged as a key modulator of plant immunity (Ballaré et al., 2012; Ballaré, 2014). There are two major pathways involved in plant immune responses: the salicylic acid (SA) pathway and the jasmonic acid (JA) pathway. The SA and JA defense pathways each induce different sets of responsive genes and are often mutually antagonistic, which enables the plant to fine-tune their defense response to a specific pathogen (Pieterse et al., 2012). The SA pathway is activated predominantly to fend off biotrophic pathogens, while the JA pathway is mainly activated in response to necrotrophic pathogens and herbivorous insects. Light is required for SA biosynthesis and activation (Genoud et al., 2002). Exposure of germinating soybean sprouts to monochromatic red light resulted in seedlings with higher levels of SA and stronger expression of SA-related defense genes that were more resistant against bacterial rotting disease (Dhakal et al., 2015). The red/far-red ratio (R:FR) is a key modulator of defense expression by which plants resolve the trade-off between resource allocation to growth or defense. Low R:FR, which signals a high risk of competition in plant canopies, represses both jasmonate-induced and salicylic acid-induced defense responses, thus redirecting resource allocation from defense to rapid plant elongation (de Wit et al., 2013; Leone et al., 2014; Ballaré and Pierik, 2017). Several studies have shown that a relatively low R:FR ratio can cause a decrease in plant resistance to necrotrophic pathogens (Cerrudo et al., 2012; Cargnel et al., 2014; Ji et al., 2019), pests (Izaguirre et al., 2006; Moreno et al., 2009), as well as biotrophic and hemibiotrophic pathogens (Shibuya et al., 2011; de Wit et al., 2013).

Apart from impacting pathogens and pests through plant-mediated effects, light can also exert a direct effect on plant attackers as well as their natural enemies. Just as plants, arthropods, and fungi possess photoreceptors, and they use light as signals for steering important developmental and/or behavioral processes (Avalos and Estrada, 2010; Johansen et al., 2011; Schumacher, 2017). Filamentous fungi maintain a complex regulatory network of photoreceptors and signal transduction pathways that enable them to use light (quantity, quality, and direction) as a signal to produce protective metabolites (e.g., carotenoids), to gear growth (direction) and development (e.g., sporulation), and to regulate their biological clock (Herrera-Estrella and Horwitz, 2007; Rodriguez-Romero et al., 2010). Arthropod photoreceptors typically have optima in the UV-A, green, and sometimes blue parts of the light spectrum (Kelber, 2001; Warrant and Nilsson, 2006). It is important to note that arthropods do not perceive light in the red and far-red part of the spectrum. Therefore, a large discrepancy exists in the perception and usage of light between arthropods and plants.

When regarding direct effects of light on arthropod pests and natural enemies, UV-A light plays a major role. Multiple studies have shown that in semi-open greenhouses that allow for UV-A to penetrate the cladding material, crops suffer from higher pest densities than in similar greenhouses with UV-absorbing cladding materials (Antignus et al., 1996; Costa and Robb, 1999; Chyzik et al., 2003; Raviv and Antignus, 2004; Legarrea et al., 2010). Direct exposure of arthropods and plant pathogens to UV-B, on the other hand, can negatively impact development and survival (Suthaparan et al., 2016; Tanaka et al., 2016; Johansen et al., 2017). Moreover, changes in light quality within the PAR spectrum can also directly affect pathogens and arthropods. The anthocorid predatory bug *Orius sauteri* developed 40 and 18% slower, respectively, and had reduced fecundity, under monochromatic red and blue light in comparison to white, green, or yellow light (Wang et al., 2013). This may have been caused by lower perceived light intensity by these arthropods due to the absence of photoreceptors in the red and blue parts of the spectrum. Indeed, the same study showed that developmental time of *O. sauteri* increased with decreasing light intensity (Wang et al., 2013). No published studies have evaluated the effect of PAR spectral composition on life history parameters and/or population growth of predatory mites. Unpublished work of Shipp, however, indicates that egg–egg development of *Amblyseius swirskii* is significantly faster under HPS supplemental lighting compared to red and blue LED lights (Buitenhuis et al., 2015). Moreover, Zilahl-Balogh et al. (2007) showed that the oviposition rate of the predatory mite *Neoseiulus cucumeris* was reduced under low light intensity, while the predation activity of this mite remained unaffected by light intensity.

In the experiments described in this paper, we grew three *S. melongena* (eggplant) genotypes under seven different LED light spectra against a background of low-intensity sunlight. We aimed to quantify the growth and development of these genotypes and tested host plant resistance against the necrotrophic pathogen *Botrytis cinerea*, the biotrophic pathogen powdery mildew (*Oidium lycopersicum*), and the herbivorous arthropod Western flower thrips (*Frankliniella occidentalis*) during both the vegetative and fruit-bearing stage. Moreover, we assessed the population growth and vertical distribution of *A. swirskii* predatory mites under a subset of four light spectra. Based on studies in the fruit vegetable crop tomato, we hypothesized that the addition of far-red light would increase stem elongation, biomass production, and assimilate partitioning toward the fruits. However, we assumed this would come at the expense of plant resistance to the abovementioned plant attackers. The expected stem elongation as a consequence of the addition of far-red light might be suppressed or overcome by decreasing the red/blue ratio. Decreasing the red/blue ratio might also reduce the expected negative effects of additional far-red light on plant resistance to pathogens and pests. Furthermore, we hypothesized that the addition of a low dose of UV-B light may increase plant resistance to pests and fungal pathogens. We expected the highest population growth of *A. swirskii* predatory mites to occur under the light spectrum with the highest proportion of green light, since mites possess photoreceptors for green light, whereas they cannot perceive light in the red part of the light spectrum. The implications of these results for greenhouse horticulture will be discussed.

## Materials and Methods

### Plant Material and Light Treatments

The experiment was conducted in a greenhouse compartment of 9.6 m × 15 m with 14 tables of 4 m × 1.8 m, each having a ceiling of dynamic LED modules (Philips GreenPower LED production modules Dynamic, Signify, Eindhoven, Netherlands). These modules are tunable in blue (B; peak at 446 nm), white [broad spectrum with large proportion of green (G) light with peak emission at 571 nm], red (R; 660 nm), and far-red (FR; 730 nm). In one of the treatments, UV light (UV; peak at 312 nm) was given at an intensity of 0.5 and 1 kJ m^–2^ day^–1^ for 30 min per day at noon during the vegetative and generative stage, respectively. The incidence of sunlight was controlled by the use of an energy screen (LS Ultra, Ludvig Svensson, Kinna, Sweden) with a transmission of 38% and a blackout screen (LS Obscura, Ludvig Svensson, Kinna, Sweden). The greenhouse was air conditioned, allowing the realization of winter conditions throughout the year. The average realized temperature was 22.0°C with a day/night temperature of 22.8 and 19.8°C, respectively, a VPD of 3.4 kPa, and a CO_2_ concentration of 631 ppm.

**FIGURE 1:**
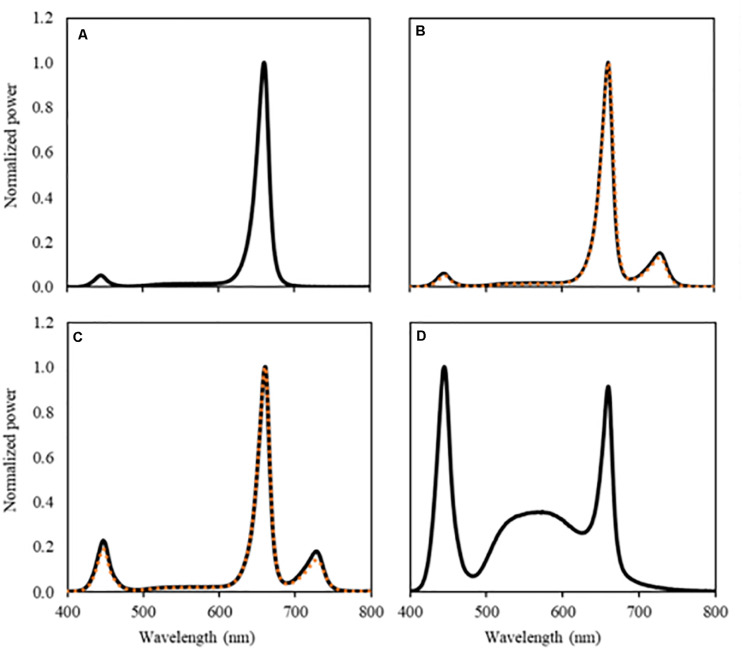
Spectral composition of the light treatments, UV-B light excluded. **(A)** RWB, **(B)** RWB FR (orange dotted line) and RWB + FR (black solid line), **(C)** RWhB FR (orange dotted line) and RWhB + FR (black solid line), and **(D)** White provided by the LEDs measured at the top of the canopy.

On July 16, 2019, eggplant cvs Tracey, Beyoncé, and Lemmy plants were transplanted on rockwool cubes (Grodan Plantop, Roermond, Netherlands) on the tables at a planting density of 20 plants/m^2^. Tracey and Beyoncé plants were sown on June 14, grafted on the rootstock Kaiser on July 3, and topped on July 15 to maintain two stems per plant. Lemmy plants were sown on June 17, grafted on July 3, and topped on July 18. The plants were placed under seven lighting strategies (Figure 1 and Table 1; one treatment per table, *n* = 2) at a light intensity of 100 μmol m^–2^ s^–1^, which was gradually increased to 140 μmol m^–2^ s^–1^ on August 30 [45 days after transplanting (DAT)] and to 180 μmol m^–2^ s^–1^ 62 DAT, at a photoperiod of 18 h. For the two treatments with additional far-red light, these PFD values were 115, 161, and 207 μmol m^–2^ s^–1^, respectively. The increase in light intensity was applied based on an expected assimilate demand based on the gradually increasing biomass of vegetative and generative organs. To prevent light pollution between treatments, tables were separated by white plastic sheets. To prevent air stratification and ensure that temperature setpoints were reached, conditioned air was distributed from top to bottom of all 14 tables through a perpendicularly located perforated sleeve. During the trial, the plants received a DLI (daily light integral) of sunlight and LED light of 8.6 mol m^–2^ s^–1^. The contributions of LED light and sunlight to the DLI were 79 and 21%, respectively, for the vegetative phase (DAT 0–24) and 88 and 12%, respectively, for the generative phase (DAT 25–90). The vegetative phase ended 24 DAT for Tracey and Beyoncé and 27 DAT for Lemmy with a final destructive harvest of three plants per table (*n* = 2, 6 plants per treatment). Thereafter, plants of the varieties Tracey and Beyoncé were placed on rockwool slabs (Grodan, Roermond, The Netherlands) at a density of three plants/m^2^ in two rows per table for the generative phase. The generative phase ended 85 DAT for variety Beyonce and 90 DAT for variety Tracey with a final destructive harvest.

**TABLE 1:** Spectral compositions of the light treatments.

Treatment	Blue (%)	Green (%)	Red (%)	Far-red (%)	PFD* (μmol m^–2^ s^–1^)
RWB	5	5	90	0	100–180
RWB UV	5	5	90	0	100–180
RWB FR	5	5	75	15	100–180
RWhB FR	15	5	65	15	100–180
RWB + FR	5	5	90	15	115–207
RWhB + FR	15	5	80	15	115–207
White	30	35	35	0	100–180

Spectral compositions consist of blue light (400–500 nm; peak at 446 nm), green light (500–600 nm, obtained by a white broadband spectrum with a large proportion of green light with peak emission at 571 nm), red light (500–600 nm; peak at 660 nm), and far-red light (700–800 nm; peak at 730 nm). In the treatment RWB UV, UV light (peak of 312 nm) is given during 30 min per day at an intensity of 0.5–1 kJ m^–2^ day^–1^. *PFD is photon flux density (μmol m^–2^ s^–1^) and was 100 μmol m^–2^ s^–1^ at transplanting (115 μmol m^–2^ s^–1^ for treatments with additional FR). PFD gradually increased to 140 μmol m^–2^ s^–1^ (161 μmol m^–2^ s^–1^ 45 DAT) and to 180 μmol m^–2^ s^–1^ (207 μmol m^–2^ s^–1^ 62 DAT).

### Plant Measurements

#### Photosynthesis and Stomatal Conductance

Light response curves of photosynthesis of Tracey leaves were measured 71 and 86 DAT in the treatments RWB and RWB + FR. Measurements were taken of four individual plants per measuring date per light treatment. The uppermost fully expanded leaf (enclosed leaf area of 6 cm^2^) was enclosed in the leaf chamber of the LI-6800 portable photosynthesis system equipped with the fluorometer chamber (LI-COR, Lincoln, NE, United States). The light spectrum during the measurements consisted of 10% blue and 90% red light. Since the light spectrum in the leaf chamber was identical for both light treatments measured, the results indicate the effects of structural changes in the leaf due to the light treatments. The block temperature was set to 25°C, CO_2_ concentration was 600 ppm, and VPD was 0.9 kPa. Light intensities applied were 1,500, 1,200, 1,000, 800, 600, 400, 300, 200, 100, 50, and 0 μmol m^–2^ s^–1^. At every light intensity, the photosynthesis, conductance, and fluorescence were measured after matching the infrared gas analyzers. The fluorescence was measured with a Multiphase protocol, with a red light target of 8,000 μmol m^–2^ s^–1^, in three phases for a total duration of 0.9 s. The quantum yield of photosystem II (ΦPSII) was calculated based on fluorescence yield data (Genty et al., 1989). The effect of light intensity on rate of photosynthesis rate, not taking into account the effect of CO_2_ concentration, can be described with a curve that reaches a plateau at high light intensity (Farquhar et al., 1980):

$$\begin{array}{c} Ass=(\varepsilon R+\left(A_{\max}+R_{d}\right)\\ -\sqrt{\left({\left(\varepsilon R+A_{\max}+R_{d}\right)}^{2}-4\Theta \varepsilon R\left(A_{\max}+R_{d}\right)\right)})/(2\Theta ) \end{array}$$

in which

*Ass*: rate of gross CO_2_ assimilation (μmol m^–2^ s^–1^)

*A*_*max*_: asymptotic value of net CO_2_ assimilation rate at high light intensity (μmol m^–2^ s^–1^)

*R*_*d*_: day respiration (μmol m^–2^ s^–1^) (in this formula, *R*_*d*_ has a negative value)

ε: initial light use efficiency (mol CO_2_ m^–2^ s^–1^/mol PAR m^–2^ s^–1^)

Θ: curvature factor

*R*: photosynthetically active radiation (μmol m^–2^ s^–1^)

Each photosynthesis light response curve was fitted to this non-rectangular hyperbola, and a two-way analysis of variance (ANOVA) with month of observation (two levels) and light treatment (two levels) was done for each of the parameters of the light response curve. In addition, a one-way ANOVA was performed for all light intensities on net assimilation rate, stomatal conductance, and ΦPSII with month of observation (two levels) as block factor.

#### Plant Morphology, Flowering, and Biomass

At the end of the vegetative phase, 24 DAT (Tracey and Beyoncé) or 27 DAT (Lemmy), three plants per table (*n* = 2; 6 plants per treatment) per variety were harvested destructively, and stem lengths, leaf area, and fresh and dry weights of stems and leaves were determined. Specific leaf area (SLA) was calculated by dividing the leaf area per plant by the dry leaf mass. In the generative phase, flowers were labeled to determine fruit growth duration (from anthesis until harvest). Ripe fruits of six plants per table were harvested twice per week in the period of 57–90 DAT (*n* = 2; 12 plants per treatment), and fresh weights and numbers of harvested fruits were recorded. Periodically, dry matter percentage of the fruits was determined by placing them in stoves at 80°C for at least 3 days. At the final destructive harvest, 90 DAT, three plants per table (*n* = 2; 6 plants per treatment) per variety were harvested destructively, and stem lengths, leaf area, and fresh and dry weights of stems, leaves, and fruits were determined. SLA was calculated by dividing the leaf area per plant by the dry leaf mass. Total fruit dry weight was calculated by multiplying the fresh weight of the harvested fruits with the dry matter percentage of these fruits and adding the dry weights of the fruits on the plant at the destructive harvest. Total shoot biomass production was calculated by adding the dry weights of the stems and leaves at the destructive harvest and the total fruit dry weight. The biomass partitioning between stems, leaves, and fruits was determined by dividing the weight of the relevant organ by the total shoot biomass. Data were analyzed by means of the statistical package GenStat (19th edition) with a two-factor ANOVA using variety and light treatments as factors. Fisher’s unprotected least significance test was used to make *post hoc* multiple comparisons among means. *P* values <0.05 were considered as significantly different for the pairwise comparisons.

### Population Development of *A. swirskii*

The population growth of *A. swirskii* predatory mites, as well as their vertical distribution on *S. melongena* plants, was assessed for a subset of four LED treatments, namely, the RWB, RWB UV, RWhB + FR, and White (see Table 1). Six of the plants on each of the two tables of the respective light treatments were used for this experiment (*n* = 2, 12 plants per treatment). One of the stems of these plants was topped to create plants with a single stem. *A. swirskii* was introduced 23 DAT at the base of the stem (80 predatory mites/plant). On the same day, as well as in each subsequent week, Typha pollen (Nutrimite^®^, Biobest) were dispersed equally over the entire plant. On 55 DAT, three leaves from each of the six plants on a table were harvested divided over three vertical layers: one leaf in layer A (1st and 2nd youngest fully grown leaves), one leaf in layer B (3rd and 4th youngest fully grown leaves), and one leaf in layer C (5th and 6th youngest fully grown leaves). On each leaf, the number of *A. swirskii* eggs, nymphs, and adults were counted (*n* = 2; 12 plants per light treatment per leaf position). The effect of light treatment and leaf position and their interaction on the number of *A. swirskii* individuals (eggs + nymphs + adults) were analyzed in Genstat 19th edition. A generalized linear mixed model with Poisson data distribution was used, and plant replicate nested within table was introduced as a random factor into the model.

### Bioassays of Host Plant Resistance

Effects on plant health were examined using leaf disc biossays with gray mold (*B. cinerea*) as a necrotroph and powdery mildew (*O. lycopersicum*) as a biotroph fungus as well as Western flower thrips (*F. occidentalis*) as a sucking insect being representatives of economically important pests and diseases in eggplant. For each table and variety, three plants were sampled (*n* = 2; 6 plants per treatment). Leaf discs with a diameter of 5 cm were taken from a medium-aged leaf (9th leaf from below) at the end of the vegetative phase as well as in the generative phase. Discs were placed on 1% water agar in 9-cm-diameter petri dishes to prevent desiccation. Subsequently, the leaf discs were inoculated with spore suspensions of 10^5^ spores/ml for botrytis obtained from a stock of strain *Botrytis* B05.10 and 10^6^ spores/ml for powdery mildew obtained from a rearing on tomato (variety Moneymaker). For the thrips bioassay, leaf discs were infested with five adult thrips each derived from a rearing on chrysanthemum (variety Miramar). The petri dishes were randomly placed in a climate room (20°C, 60% RH, fluorescent tubes 23 μmol m^–2^ s^–1^). Four days after inoculation, incidence of *B. cinerea* was measured as diameter of necrotic lesions (mm^2^). Incidence of powdery mildew was measured 10 days after inoculation as Spencer index with a range of 1–5 whereby 0 represents no infection and 1, 2, 3, 4, and 5 represent 1, 2–5, 6–20, 20–40, and >40% infection, respectively. Toward the end of the experiment, a natural infection with powdery mildew occurred. Therefore, at the end of the experiment, four whole plants per table and variety were scored for mildew infection using the Spencer index (*n* = 2, 8 plants per treatment). Thrips damage was scored as silver damage (mm^2^) 5 days after infestation. Data for *B. cinerea* were analyzed with a two-factor ANOVA using variety and light treatments as factors, whereas data for powdery mildew were analyzed with a χ^2^ test.

## Results

### Plant Growth, Development, and Biomass Partitioning

At the end of the vegetative phase, plants that received far-red light were taller than the treatments without far-red light (Table 2). Total plant dry weight was higher for the treatments with additional far-red light (RWB + FR and RWhB + FR), primarily due to the increased leaf dry weight. Leaf area did not differ between treatments for the varieties Tracey and Beyoncé, whereas in Lemmy, leaf area was higher (Table 2). SLA was lower for the treatments with additional far-red light, implying that they are capable of intercepting more light with a comparable leaf biomass.

**TABLE 2:** Effects of spectral composition on vegetative eggplant traits.

Variety treatment	Stem length (cm)	Leaf dry weight (g plant^–1^)	Stem dry weight (g plant^–1^)	Shoot dry weight (g plant^–1^)	Leaf area (10^–3^ m^2^ plant^–1^)	SLA (m^2^ kg^–1^)
**Tracey**						
RWB	10.7 b	7.4 b	0.9 c	8.2 b	2.4 a	32.9 a
RWB UV	11.2 b	8.0 b	1.0 c	8.9 b	2.5 a	33.0 a
RWB FR	19.2 a	7.7 b	1.6 ab	9.3 b	2.4 a	32.5 a
RWhB FR	18.6 a	6.3 b	1.3 b	7.6 b	2.2 a	35.9 a
RWB + FR	17.3 a	9.4 a	1.9 a	11.3 a	2.5 a	26.2 b
RWhB + FR	18.0 a	9.8 a	1.6 ab	11.4 a	2.6 a	25.2 b
White	10.3 b	7.7 b	0.9 c	8.7 b	2.6 a	32.1 a
**Beyoncé**						
RWB	12.3 b	7.8 c	0.8 b	8.6 b	2.5 a	34.3 a
RWB UV	10.6 b	8.1 c	1.0 b	9.1 b	2.5 a	31.8 ab
RWB FR	19.3 a	8.0 c	1.5 a	9.5 b	2.5 a	30.2 bc
RWhB FR	17.8 a	8.5 c	1.5 a	9.9 b	2.6 a	29.9 bc
RWB + FR	18.3 a	10.5 a	1.8 a	12.3 a	2.6 a	24.0 d
RWhB + FR	17.3 a	9.9 ab	1.6 a	11.5 a	2.6 a	27.5 cd
White	11.9 b	8.6 bc	1.0 b	9.6 b	2.6 a	29.7 bc
**Lemmy**						
RWB	10.7 c	8.4 a	1.0 b	9.4 a	2.6 a	32.4 b
RWB UV	10.3 c	6.6 bc	0.8 b	7.4 b	2.2 b	36.3 a
RWB FR	22.0 a	6.1 c	1.4 a	7.5 b	2.1 b	36.5 a
RWhB FR	21.1 a	6.5 bc	1.5 a	7.9 ab	2.4 ab	38.1 a
RWB + FR	18.9 b	7.6 ab	1.7 a	9.3 a	2.4 ab	32.2 b
RWhB + FR	19.3 b	7.6 ab	1.6 a	9.2 a	2.5 ab	32.8 b
White	11.8 c	6.3 bc	0.9 b	7.2 b	2.2 b	36.9 a
**Two-way ANOVA**						
Treatment	***	***	***	***	n.s.	***
Variety	n.s.	***	n.s.	***	*	***
Treatment × variety	n.s.	*	n.s.	n.s.	n.s.	n.s.

Light treatments (see Table 1) were maintained during 25 days on stem length (average length of two stems per plant), shoot dry weight (sum of stem dry weight and leaf dry weight per shoot), leaf area per shoot, and specific leaf area, calculated as the leaf dry weight divided by the leaf area (n = 2, 6 plants per treatment). Different letters per variety within columns indicate significant differences (P < 0.05). The means are tested with two-way analysis of variance (ANOVA) for significant effects of treatment, variety, and the interaction between treatment and variety. Fisher’s unprotected least significance test was used to make post hoc multiple comparisons among means. Significant differences are indicated: *P < 0.05; ***P < 0.001; n.s., non-significant.

Total fresh fruit production (kg m^–2^) in the generative phase for both varieties was highest for the treatment RWB + FR, followed by RWhB + FR (Table 3). Fruit production in the treatments where far-red light was given at the expense of PAR light (RWB FR and RWhB FR) also had a higher production than the reference treatment. Production differences were due to the total number of harvested fruits and the average fruit fresh weight (g fruit^–1^). Fruit growth duration from anthesis to harvest in the treatments White and RWB ranged from 21 days for Beyoncé to 23–25 days for Tracey (Table 3). In the treatments with additional far-red light, fruit growth duration was shortened by 4 to 7 days. The number of harvested fruits was highest in RWB + FR, followed by RWhB + FR, for both varieties (Table 3). In the treatments where far-red replaced part of the PAR light (RWB FR and RWhB FR), the number of harvested fruits was lower than when far-red light was given additionally. The number of fruits harvested was lowest in the reference treatment (RWB), as well as the average fruit weight (Table 3). Fruits from the treatments with far-red FR light had the highest dry matter percentage (Table 3), although the dry matter percentage of fruits from RWhB FR did not differ significantly from the treatments RWB, RWB UV, and White. As at the end of the vegetative phase, stem length at the end of the generative phase was highest for the treatments with far-red light (data not shown). Leaf area or SLA differed between treatments for either variety (*P* = 0.20 and *P* = 0.44, respectively; data not shown). At the final destructive harvest, dry weights of the stems, leaves, and fruits on the plants were determined, and the dry weights of the harvested fruits (calculated by FW × dry matter percentage of a sample of fruits) were added to determine total shoot biomass. Total shoot dry weight was highest for the treatments with additional far-red light (RWB + FR and FWhB + FR), followed by the other treatments with far-red light in the spectrum (RWB FR, RWhB FR, and White). Treatments with far-red partitioned a larger part of their biomass into the fruits, at the expense of the leaves (Table 4).

**TABLE 3:** Effects of spectral composition on fruit production of eggplant.

Variety treatment	Fresh fruit production (kg m^–2^)	Fruit growth duration (days) plant^–1^)	Number of harvested fruits (-)	Average fruit weight (g fruit^–1^)	Dry matter percentage of the harvested fruits (%)
**Tracey**					
RWB	3.2 d	23 ab	40 c	254 b	4.9 b
RWB UV	3.4 d	24 ab	40 c	269 ab	4.9 b
RWB FR	4.4 b	21 ab	49 b	286 a	5.2 ab
RWhB FR	4.5 b	20 b	49 b	293 a	5.3 a
RWB + FR	5.2 a	20 b	57 a	290 a	5.1 ab
RWhB + FR	4.9 ab	18 c	52 b	300 a	5.3 a
White	3.8 c	25 a	43 c	280 ab	5.0 ab
**Beyoncé**					
RWB	2.9 e	21 ab	33 d	279 c	4.9 b
RWB UV	3.3 d	23 a	37 c	289 bc	5.0 b
RWB FR	4.2 c	18 bc	44 b	300 bc	5.3 a
RWhB FR	4.2 c	18 bc	44 b	302 bc	5.0 ab
RWB + FR	5.5 a	19 abc	55 a	318 a	5.3 a
RWhB + FR	5.0 b	17 c	51 a	310 ab	5.2 ab
White	3.3 de	21 ab	37 c	284 bc	5.1 ab
**Two-way ANOVA**					
Treatment	***	**	***	**	*
Variety	n.s.	*	***	**	n.s.
Treatment × variety	n.s.	n.s.	n.s.	n.s.	n.s.

Light treatment (see Table 1) effects on fresh fruit production (kg m^–2^) in the period of 57 to 90 days after transplanting for the varieties Tracey and Beyoncé (n = 2, measured on 12 plants per treatment). Different letters per variety within columns indicate significant differences (P < 0.05). The means are tested with two-way analysis of variance (ANOVA) for significant effects of treatment, variety, and the interaction between treatment and variety. Fisher’s unprotected least significance test was used to make post hoc multiple comparisons among means. Significant differences are indicated: *P < 0.05; **P < 0.01; ***P < 0.001; n.s., non-significant.

**TABLE 4:** Effects of spectral composition on assimilate partitioning of eggplant.

Variety	Treatment	Shoot biomass (g plant^–1^)	Partitioning to the leaves (%)	Partitioning to the stem (%)	Partitioning to the fruits (%)
Beyoncé	RWB	157 d	43 a	15 a	42 b
	RWB UV	181 cd	42 a	15 a	43 b
	RWB FR	198 bc	32 b	17 a	51 a
	RWhB FR	193 c	33 b	17 a	50 a
	RWB + FR	241 a	30 b	16 a	54 a
	RWhB + FR	222 ab	31 b	16 a	52 a
	White	174 cd	40 a	17 a	43 b
Tracey	RWB	159 d	38 a	16 bc	45 b
	RWB UV	165 d	39 a	16 c	45 b
	RWB FR	203 bc	31 b	17 bc	52 a
	RWhB FR	201 bc	28 b	20 a	52 a
	RWB + FR	218 ab	30 b	18 abc	52 a
	RWhB + FR	229 a	31 b	19 ab	50 a
	White	180 cd	38 a	17 bc	45 b
Two-way ANOVA	Treatment	***	***	*	***
	Variety	n.s.	**	**	n.s.
	Treatment × variety	n.s.	n.s.	n.s.	n.s.

Effect of spectral composition on total shoot dry weight (including harvested fruits) at the destructive harvest (90 days after transplanting) and assimilate partitioning over leaves, stems, and fruits for the varieties Tracey and Beyoncé (n = 2; measured on 6 plants per treatment). Different letters per variety per column indicate statistical differences. The means are tested with two-way analysis of variance (ANOVA) for significant effects of treatment, variety, and the interaction between treatment and variety. Fisher’s unprotected least significance test was used to make post hoc multiple comparisons among means. Significant differences are indicated: *P < 0.05; **P < 0.01; ***P < 0.001; n.s., non-significant.

**FIGURE 2:**
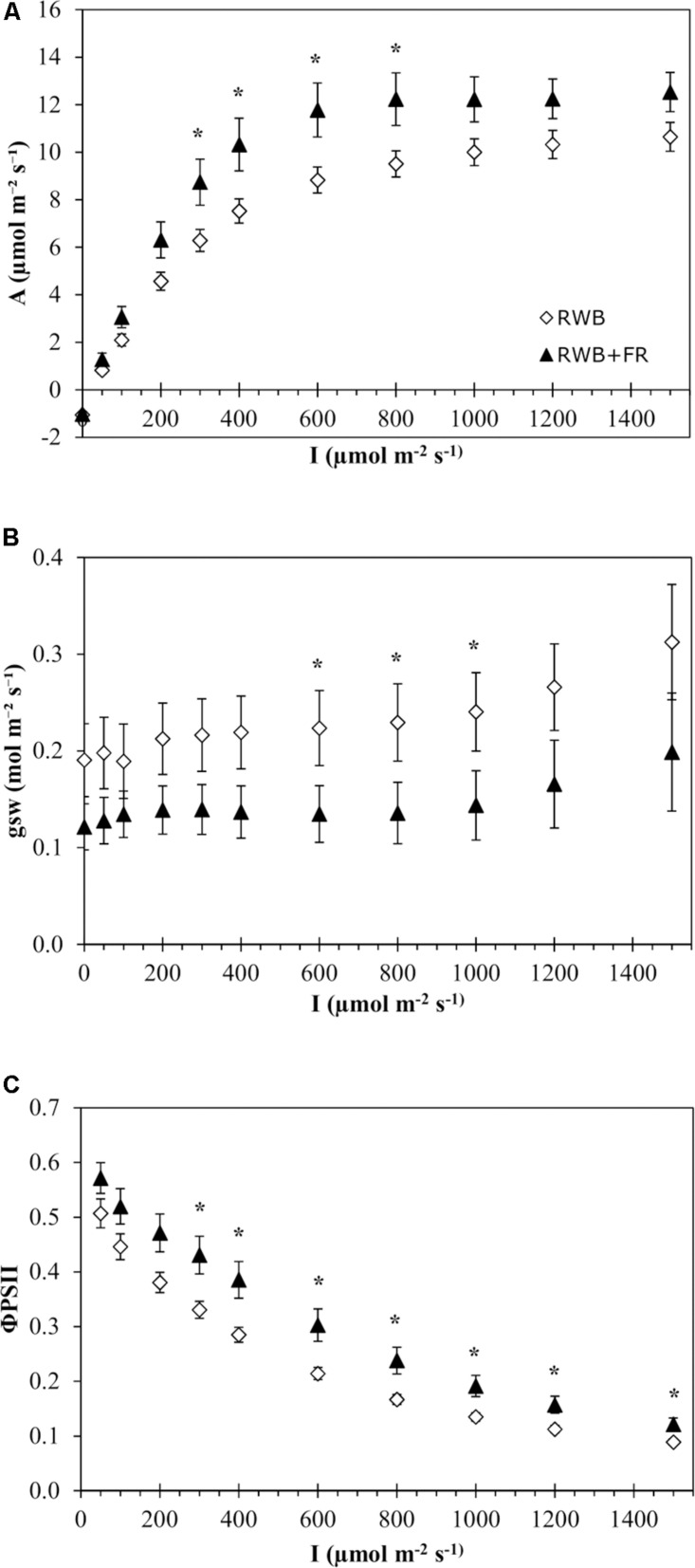
Effect of the light treatments RWB and RWB + FR on the pattern of **(A)** net photosynthetic response, **(B)** stomatal conductance, and **(C)** ΦPSII to a series of light intensities (0–1,500 μmol m^– 2^ s^– 1^) of the uppermost unshaded leaves of *Solanum melongena*, variety Tracey. Each data point represents the mean of two series of measurements on four individual plants per treatment (*n* = 2; 8 plants per treatment) ± standard error of the mean. Data were analyzed with a one-way ANOVA for all light month of observation (two levels) as block factor. Significant differences (*P* ≤ 0.05) between treatments are indicated with asterisk (*).

Analysis of the light response curves of photosynthesis for the variety Tracey showed that the addition of far-red light to the RWB spectrum did not have a significant effect on any of the four parameters describing the non-rectangular hyperbolas (*P* = 0.49 for *A*_*max*_, *P* = 0.69 for *R*_*d*_, *P* = 0.44 for ε, and *P* = 0.19 for Θ; Figure 2A). Comparisons between treatments per individually measured light intensity showed significant differences in rate of photosynthesis between 300 and 800 μmol m^–2^ s^–1^ (*P* = 0.041). Stomatal conductance was lower in the treatment with additional far-red light between 600 and 1,000 μmol m^–2^ s^–1^ (*P* = 0.047) (Figure 2B). ΦPSII efficiency was higher in the treatment with additional far-red light (Figure 2C). Between 300 and 1,500 μmol m^–2^ s^–1^, ΦPSII was up to 43% higher in the treatment RWB + FR compared to RWB (*P* = 0.019). The observed rates of photosynthesis are low, which might be due to the varying levels of chlorosis in the leaves during measurements. Between 600 and 800 μmol m^–2^ s^–1^, stomatal conductance was higher in the RWB treatment compared to RWB + FR treatment, but assimilation rates were lower, indicating that conductance was not limiting assimilation rate.

### Population Development of *A. swirskii*

**FIGURE 3:**
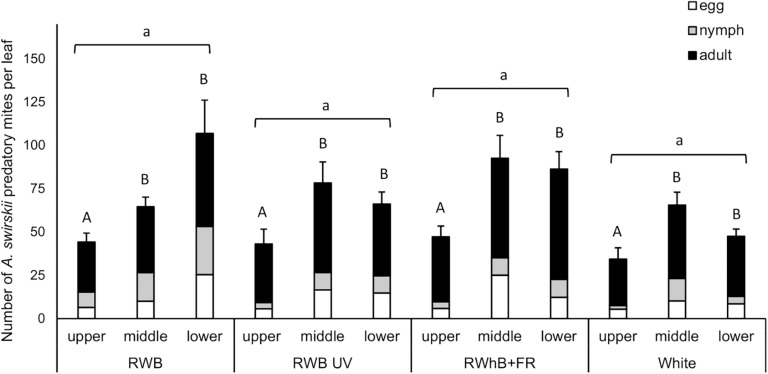
Effect of the light treatments RWB, RWB UV, RWhB + FR, and White on the average number of *Amblyseius swirskii* individuals per leaf (± standard error of the mean), subdivided into average numbers of eggs, nymphs, and adults, in the upper, middle, and lower leaf layers (*n* = 2; 12 plants per treatment). Data were analyzed with a generalized linear mixed model with Poisson data distribution where plant replicate nested within table was introduced as a random factor into the model. Different small letters indicate significant differences (*P* < 0.05) between light treatments and different capital letters indicate significant differences (*P* < 0.05) between leaf layers.

The light treatments did not significantly affect population size of *A. swirskii*, measured as number of individuals (eggs + nymphs + adults) per leaf, at the time of evaluation (*P* = 0.99; Figure 3). Leaf position, however, did affect the population size (*P* < 0.001), with lower numbers of predatory mites per leaf on the leaves of the upper leaf layer (on average 42 individuals/leaf) compared to the leaves of the middle and lower leaf layers (on average 74 and 73 mites/leaf, respectively). The difference in *A. swirskii* number between leaf layers did not depend on the light treatment (*P* = 0.22).

### Host Plant Resistance Against Botrytis, Powdery Mildew, and Thrips

**FIGURE 4:**
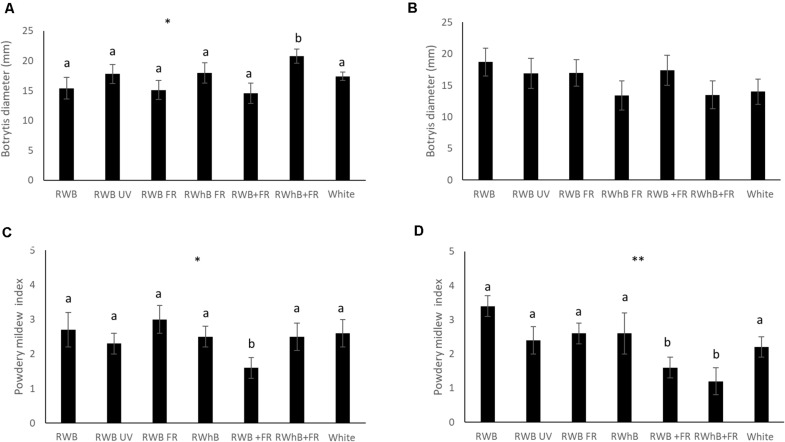
Effect of spectral composition on eggplant host plant resistance (varieties Tracy, Beyoncé, and Lemmy) to *B. cinerea* in panel **(A)** the vegetative and **(B)** the generative phase, and to powdery mildew (*Oidium lycopersicum*) in panel **(C)** the vegetative and **(D)** the generative phase. Data represent the mean (*n* = 2, 6 plants per treatment per variety) ± standard error of the mean. Data were analyzed with ANOVA for botrytis and with a χ^2^ test for powdery mildew. Different letters denote significant differences between treatments at *P ≤ 0.05 and **P ≤ 0.01.

For the vegetative phase, no differences between varieties in infestation with *B. cinerea* or powdery mildew could be observed (data not shown). Thrips damage, however, was significantly higher in the variety Lemmy (average area of 50 ± 4 mm^2^) than in the varieties Tracy and Beyoncé with average areas of 39 ± 3 mm^2^ and 38 ± 4 mm^2^, respectively (*P* = 0.04; data not shown). In the generative phase, no differences in incidence of *B. cinerea* or thrips infestation occurred between varieties (data not shown). However, the area infected by powdery mildew in the variety Tracey was three times larger compared to Beyoncé (*P* ≤ 0.001; data not shown). Infection of *B. cinerea* in the vegetative phase was significantly higher under RWhB + FR (*P* = 0.05) compared to the other light treatments (Figure 4A), but did not differ in the generative phase (Figure 4B). In contrast, infection of powdery mildew was lower (*P* = 0.05) under RWB + FR in the vegetative phase (Figure 4C) and under RWB + FR as well as RWhB + FR (*P* = 0.007) in the generative phase (Figure 4D). Infection of powdery mildew on whole plants that were naturally infected was strongly reduced under RWB UV (*P* ≤ 0.001; data not shown). Thrips damage was not different between light treatments in the vegetative and the generative phase (data not shown).

## Discussion

In this study, the effects of a range of spectral compositions of LED light on plant growth and development, plant resilience to fungal diseases, and the establishment of *A. swirskii* predatory mites were quantified. Plant biomass and fruit production were favorably affected by increasing the contribution of far-red light in the spectrum, which was only moderately compensated for by an increased susceptibility to fungal pathogens. The observations underlying these conclusions and the implications of these results are discussed below.

### Additional Far-Red Increases Biomass and Partitioning to the Fruits

Providing additional far-red light in eggplant increased fruit fresh production, total biomass, and partitioning to the fruits, in accordance with earlier findings in tomato (Ji et al., 2019; Kalaitzoglou et al., 2019). In the vegetative stage, additional FR resulted in longer stems and a more open plant structure (Table 2). This may have positively affected light interception and thereby crop photosynthesis, as indicated by 3D model simulations (Sarlikioti et al., 2011). Indeed, total shoot biomass at the end of the vegetative phase was increased by additional far-red light (Table 2), which corroborates findings of Park and Runkle (2017). In the generative phase, total fruit production increased when additional far-red light was supplied. The increased fraction of dry matter partitioned to the fruits was primarily due to the increased number of fruits and the shorter fruit growth duration. This is in accordance with general effects of far-red light on flowering (Demotes-Mainard et al., 2016) and fruit set (Ji et al., 2019). Exposure to far-red light may increase the efficiency of photosystem II electron transport by balancing the excitation of both photosystems, thereby increasing the net photosynthesis rate. This effect has been described as the Emerson enhancement effect (Emerson et al., 1957; Zhen and van Iersel, 2017). This effect directly contributes to rate of leaf photosynthesis during the hours of the day that no natural far-red light from the sun is present, which in our experiment was between midnight and 8:30 a.m. This effect was quantified by Kalaitzoglou et al. (2019), who reported an increase in rate of photosynthesis of 15% when 54 μmol m^–2^ s^–1^ far-red was added to a red/blue spectrum. However, in our trial, only indirect effects of the light spectra on rate of photosynthesis were determined, since photosynthesis measurements were performed under a fixed red/blue spectrum in the leaf chamber of the LI-6800. We observed a positive trend of the far-red light treatment on the capacity for CO_2_ fixation per unit leaf area (Figure 2), although none of the parameters of the photosynthesis response curves that were fitted were significantly different. However, when net assimilation rate was analyzed per light intensity measured, rates were significantly higher in the RWB + FR treatment compared to the reference (RWB), between 300 and 800 μmol m^–2^ s^–1^. Since far-red photons preferentially excite PSI reaction centers, leaf acclimation can result in an increased PSII/PSI ratio (Hogewoning et al., 2012). Indeed, stomatal conductance was not different between treatments, but the ΦPSII was higher for the treatment with additional far-red light, indicating acclimation of leaf photosystem composition. However, the net assimilation rates were low for both treatments. Romanatti et al. (2019) reported an assimilation rate of 15.1 μmol CO_2_ m^–2^ s^–1^ at a light intensity of 700 μmol m^–2^ s^–1^ and 390ppm CO_2_, whereas we measured 12.0 and 9.0 μmol CO_2_ m^–2^ s^–1^ at a light intensity of 800 μmol m^–2^ s^–1^ for the RWB + FR and RWB treatments, respectively. This indicates that the photosynthetic performance under the LED treatments was suboptimal and further research toward a sustainable cultivation method that includes LED lighting is required.

So far, all studies in which the effects of altered R:FR ratios in horticultural systems were examined added additional far-red light to the PAR light sum (Ji et al., 2019; Kalaitzoglou et al., 2019; Zhang et al., 2019; Zhen and Bugbee, 2020). When additional far-red light is supplied, the total photon flux (PDF) increases, as well as the electricity consumption of the lighting system. From a sustainability perspective, it would be relevant to know the effects of a spectrum that includes far-red light compared to a reference spectrum with the same PFD. Therefore, we investigated the effects of replacing part of the PAR light by far-red on plant growth and crop resilience, while maintaining PFD. In the vegetative phase, replacing part of the PAR light by far-red resulted in lower leaf and shoot dry weights compared to the treatments with additional far-red, due to the lower level of PAR light. However, direct shade avoidance effects such as plant height and leaf size (Evans and Poorter, 2001) did not differ between the treatments with additional far-red light or in which far-red replaced part of the PAR light. In spite of the lower level of PAR light, the treatments where far-red replaced part of the PAR light (RWB FR and RWhB FR), shoot biomass was comparable to the reference, indicating that far-red light positively affected light interception and thereby crop photosynthesis. In the generative phase, shoot biomass in the treatments where part of the PAR light was replaced by far-red was lower than in the treatments with additional far-red light, although the biomass partitioning between leaves, stems, and fruits was comparable (Table 3). Early fruit production in the treatments where PAR was partly replaced by far-red light was 45% higher than in the reference treatment. This shows that altering the spectral composition of the light while maintaining the PFD is favorable for biomass accumulation and fruit production. This offers perspectives in the design of energy-efficient greenhouse cultivation systems.

In general, decreased ratios of red to blue radiation reduce biomass accumulation and extension growth, but enhance pigmentation and nutritional value (Huché- Thélier et al., 2016), although growth responses to R:B can vary depending on species (Hernández and Kubota, 2016). In our study, we applied two levels of red/blue ratio, assuming that the increased percentage of blue light might counteract some of the far-red light effects (Runkle and Heins, 2001). However, increasing the percentage of blue light in the presence of far-red light did not have any effect on plant height or shoot weight in the vegetative phase, which is in agreement with findings of Meng and Runkle (2019) for shoot dry weight and hypocotyl length in lettuce and basil. In the generative phase, a higher percentage of blue light reduced the fruit growth duration and increased the fruit production only in the treatments with additional far-red (RWhB + FR compared to RWB + FR), thereby indicating an interaction with PAR levels. Kaiser et al. (2019a) also reported a favorable effect of blue light on fruit production, with an optimum of 6–12%. In line with findings in tomato (Kaiser et al., 2019b), the white light treatment containing a higher proportion of green light showed a tendency toward increased biomass production compared to the reference treatment.

### Light Spectrum Affects Host Plant Resistance to Pathogens

In all eggplant genotypes, the treatments with far-red light induced shade avoidance responses such as increased stem length and led to a higher shoot biomass and fruit production. However, the treatments with far-red light did not show negative effects on host plant resistance to *B. cinerea*, except for the vegetative plants under high blue and additional far-red light (RWhB + FR). It seems that more resource allocation to growth in vegetative eggplant only marginally compromised allocation to defense, while it did not affect fruit-bearing eggplant at all. This is in contrast to the findings in Arabidopsis (Cerrudo et al., 2012; Cargnel et al., 2014) and tomato (Ji et al., 2019) where low R:FR repressed jasmonate-induced defense leading to increased *B. cinerea* infection. While vegetative Arabidopsis rosettes were subjected to a R:FR ratio of 0.55, environmental factors including R:FR ratio, PAR level, and photoperiod used in tomato (Ji et al., 2019) were comparable to our study. Effects of low R:FR on defense may thus possibly depend on plant species. For both genotypes and growth stages, additional far-red light increased plant resistance to powdery mildew as a biotroph, irrespective of the proportion of blue light. This effect was not observed in the treatments where far-red light replaced part of the PAR light, which resulted in lower PAR levels than in the treatments with additional far-red light. Lower PAR can potentially lead to lower host plant resistance as has been shown in tomato (Escobar-Bravo et al., 2018). Opposite to our results, de Wit et al. (2013) observed that low R:FR ratio enhanced susceptibility to the hemibiotroph pathogen *Pseudomonas syringae* in Arabidopsis. There is, however, one major difference between their experimental setup and ours. In our study, exposure of the plants to the light treatments preceded the inoculation with the pathogen, while in the study of de Wit et al. (2013), inoculation of the pathogen preceded the exposure of plants to the light treatments. This difference in the order in which plants were challenged by pathogen attack and low R:FR light treatment may be part of the explanation why a low R:FR light treatment led to these opposite results. Our findings that low R:FR ratio marginally restrained host plant resistance to *B. cinerea* while resistance to powdery mildew was increased may be explained by a shift between the mutually antagonistic JA and SA defense pathways. Gene expression studies of major SA- and JA-dependent genes in response to low R:FR treatments would be useful for subsequent investigations of host plant resistance in eggplant. Meanwhile, our results point out that altering the spectral composition of LED light could offer the potential to not only increase plant growth and production but also enhance host plant resistance to diseases.

Treatment with UV-B did not increase plant resistance to powdery mildew, but seemed to have a strong direct effect on this pathogen. This observation, however, was based on a spontaneous natural mildew infection without standardized inoculation. Direct effects of UV-B on mildew have been reported in cucumber (Suthaparan et al., 2014) and tomato (Suthaparan et al., 2016). Powdery mildew is an ecto-parasite, whose colorless hyphae without pigmentation are likely to be especially vulnerable to UV treatment. Plants treated with additional far-red light did not show reduction of powdery mildew infection, although the leaf bioassays had shown enhanced host plant resistance. Possibly, the natural infection was much stronger than the inoculated one, diminishing any plant resistance effect. Host plant resistance to thrips or *B. cinerea* was affected by UV-B treatment. A strong reduction of thrips silver damage was reported by Escobar-Bravo et al. (2019a) using the same UV-B intensity in tomato as in our trial. Doubling of this UV-B intensity was needed to increase resistance to thrips in chrysanthemum whereby effects were genotype dependent (Escobar-Bravo et al., 2019b). Although eggplant belongs to the plant family of the Solanaceae, as does tomato, it appears that UV-B treatments to increase host plant resistance may be species specific, especially seen in the relatively low window of effective UV-B ranges (Escobar-Bravo et al., 2019a). This is supported by the fact the UV-B intensity used for increasing plant resistance to *B. cinerea* in Arabidopsis was 18-fold higher compared to the one used in tomato (Demkura and Ballaré, 2012).

### Light Spectrum Does Not Affect Population Development of *A. swirskii*

In this study, no effect of LED spectrum on population growth of *A. swirskii* could be observed. This is contrary to our hypothesis that *A. swirskii* would reproduce faster under the light spectrum with the highest proportion of green light, which was based on the premise that predatory mites can only perceive light in the green and UV-A parts of the spectrum. It differs from the results of the unpublished work of Shipp that indicates that *A. swirskii* develops faster under supplemental lighting with HPS lamps compared to red and blue LED lights (Buitenhuis et al., 2015). It also contrasts the work of Wang et al. (2013) on the predatory bug *O. sauteri* that shows that the developmental time of *O. sauteri* increased and fecundity decreased under monochromatic red light compared to white light. In our study, however, LED spectra were supplied against a background of low-intensity natural sunlight, whereas Wang et al. (2013) used monochromatic LED treatments and Buitenhuis et al. (2015) did not mention the conditions under which the study of Shipp was performed. Whether the lack of an influence of light spectrum against a background of low-intensity sunlight on the population growth of *A. swirskii* can also be extrapolated to other climate conditions or predatory mite species remains to be investigated. The study of Zilahl-Balogh et al. (2007) showed that oviposition rate of the predatory mite *Amblyseius cucumeris* was reduced under low light intensity, and this effect tended to be more pronounced under lower temperature (20°C) and short day conditions (8 h light) than at higher temperature (24°C) and long day conditions (16 h). We can therefore not exclude the possibility that the effect of light spectrum on *A. swirskii* is dependent on the temperature and daylength conditions. UV-B irradiation has been previously observed to exert an inhibiting effect on the population growth of spider mites (Tanaka et al., 2016). A major difference between the study of Tanaka et al. (2016) and our study is the use of light reflection sheets that increased the UV-B irradiation on the abaxial side of the leaves. In our study, UV-B only reached the plants from above, thus allowing *A. swirskii* to hide from the UV-B irradiation. Moreover, the light spectra tested did not exert any effect on the vertical distribution of these predatory mites over the crop. In all four light treatments, less mites were present on the upper leaf layer compared to the lower leaf layers, which is in accordance with the findings of Messelink et al. (2006).

### Conclusion

Our results demonstrate that including far-red light in the supplementary lighting spectrum significantly increased eggplant fruit fresh and dry mass production. This increase was due to the increase of the fraction of dry mass partitioned to the fruits, due to the reduced fruit growth duration and increased number of harvested fruits. A crucial element in the design of sustainable LED lighting strategies for greenhouse horticulture is their effect on pathogens, pests, and their natural enemies. Adding far-red light to the spectrum reduced host plant resistance to *Botrytis*, but increased the resistance to powdery mildew. No effects of light spectrum on the population development of *A. swirskii* or resistance to thrips were observed. These combined results indicate that LED lighting strategies should be developed for crops, depending on the major pathogens and pests occurring in those crops.

## Data Availability Statement

The raw data supporting the conclusions of this article will be made available by the authors, without undue reservation.

## Author Contributions

JA, KL, HM, and KW: conception and design of the experiments. KW, HM, and KL: execution of the experiments and analysis of the data. JA, HM, and KL: writing of the manuscript. All authors contributed to the article and approved the submitted version.

## Conflict of Interest

The authors declare that the research was conducted in the absence of any commercial or financial relationships that could be construed as a potential conflict of interest.
